# Modeling a Nociceptive Neuro-Immune Synapse Activated by ATP and 5-HT in Meninges: Novel Clues on Transduction of Chemical Signals Into Persistent or Rhythmic Neuronal Firing

**DOI:** 10.3389/fncel.2020.00135

**Published:** 2020-05-19

**Authors:** Alina Suleimanova, Max Talanov, Oleg Gafurov, Fail’ Gafarov, Ksenia Koroleva, Anaïs Virenque, Francesco M. Noe, Nikita Mikhailov, Andrea Nistri, Rashid Giniatullin

**Affiliations:** ^1^Laboratory of Neurobiology, Kazan Federal University, Kazan, Russia; ^2^Neuroscience Center, Helsinki University, Helsinki, Finland; ^3^A.I. Virtanen Institute for Molecular Sciences, University of Eastern Finland, Kuopio, Finland; ^4^Department of Neuroscience, International School for Advanced Studies, Trieste, Italy

**Keywords:** model, migraine, trigeminal nerve, mast cells, ATP, 5-HT

## Abstract

Extracellular ATP and serotonin (5-HT) are powerful triggers of nociceptive firing in the meninges, a process supporting headache and whose cellular mechanisms are incompletely understood. The current study aimed to develop, with the neurosimulator NEURON, a novel approach to explore in silico the molecular determinants of the long-lasting, pulsatile nature of migraine attacks. The present model included ATP and 5-HT release, ATP diffusion and hydrolysis, 5-HT uptake, differential activation of ATP P2X or 5-HT3 receptors, and receptor subtype-specific desensitization. The model also tested the role of branched meningeal fibers with multiple release sites. Spike generation and propagation were simulated using variable contribution by potassium and sodium channels in a multi-compartment fiber environment. Multiple factors appeared important to ensure prolonged nociceptive firing potentially relevant to long-lasting pain. Crucial roles were observed in: (i) co-expression of ATP P2X2 and P2X3 receptor subunits; (ii) intrinsic activation/inactivation properties of sodium Nav1.8 channels; and (iii) temporal and spatial distribution of ATP/5-HT release sites along the branches of trigeminal nerve fibers. Based on these factors we could obtain either persistent activation of nociceptive firing or its periodic bursting mimicking the pulsating nature of pain. In summary, our model proposes a novel tool for the exploration of peripheral nociception to test the contribution of clinically relevant factors to headache including migraine pain.

## Introduction

The mechanisms responsible for migraine pain, a common and devastating condition, remain poorly understood. Nevertheless, the prevailing opinion suggests that one major component of migraine pain originates from the trigeminal nerve fibers located in meningeal tissues to send nociceptive signals to brainstem nuclei (Messlinger, 2009; Olesen et al., 2009; Pietrobon and Moskowitz, 2013; Zakharov et al., 2015). The meninges harbor a large population of mast cells, which can release various chemicals that activate nearby nerve fibers (Levy et al., 2007; Theoharides et al., 2007; Kilinc et al., 2017). According to the original purinergic hypothesis of migraine (Burnstock, 1981), extracellular ATP (eATP) is one such player for the onset of migraine pathology. In fact, in addition to the effect of eATP on blood vessels (Burnstock, 1981), this endogenous purine can interact with pro-nociceptive P2X3 receptors located almost exclusively on sensory neurons (Cockayne et al., 2000; Souslova et al., 2000), indicating P2X3 receptors as important contributors to pain generation.

Previous studies have described the unique properties of P2X3 receptors including fast activation and desensitization and slow rate of resensitization (Chen et al., 1995; Lewis et al., 1995; Vulchanova et al., 1996; Jin et al., 2004; Burnstock, 2008; Giniatullin and Nistri, 2013). To describe these characteristics, we developed a cyclic model for the function of P2X3 receptors that closely replicates P2X3 mediated responses (Sokolova et al., 2006). However, the original data used for modeling were obtained from cultured sensory neurons and recombinant receptor systems, leaving open the question of their *in vivo* applicability. One paradox (North, 2004) that remains unsolved is how the strong desensitization of P2X3 receptors commonly observed with a patch-clamp recording from cultured neurons is compatible with the well-known role of this ATP-driven receptor in sustained pain signaling (Cockayne et al., 2000; Souslova et al., 2000).

Our recent work has further supported the purinergic hypothesis of migraine by showing the ability of ATP and its chemical analogs to trigger persistent spiking in trigeminal nerve fibers present in the whole-mount rat meninges (Yegutkin et al., 2016). Furthermore, using mast cell-deficient mice, we have shown that eATP could activate trigeminal nerves both directly as well as *via* release of 5-hydroxytryptamine (5-HT) originating from degranulation by immune cells (Koroleva et al., 2019). Interestingly, 5-HT is not only a powerful trigger for prolonged nociceptive firing in meningeal afferents (Kilinc et al., 2017) but also a well-known sensitizing agent (Vaughn and Gold, 2010).

The complex interplay among ATP, 5-HT, and their mast cell release process remains, however, to be elucidated. To address this complex phenomenon, the present study applied a modeling approach to explore the impact of ATP and 5-HT release from immune cells (meningeal mast cells), ATP hydrolysis and diffusion, 5-HT uptake, ATP-activated P2X3 (Sokolova et al., 2006) and P2X2 receptors (Simonetti et al., 2006; Moffatt and Hume, 2007), and of 5-HT-activated 5-HT3 receptors (Corradi et al., 2009). In addition to the standard role of sodium and potassium channels in membrane excitability, former modeling studies have highlighted the importance of certain subtypes of the sodium channel in coding sensory information by nociceptive sensory neurons. Thus, one computational model has described their role in sensory signaling by dorsal DRG neurons innervating the urinary bladder (Mandge and Manchanda, 2018). Zhao et al. (2016) have shown that the density of sodium channels determines the fidelity and precision of neuronal sensory coding. Likewise, the model of C-fibers by Tigerholm et al. (2014) has shown the characteristics of axonal spike propagation in human C-nociceptors. Whereas several subtypes of sodium channel are expressed by nociceptive neurons, the subtypes Nav1.7 and Nav1.8 play are particularly important ones for generation and propagation of action potentials (Choi and Waxman, 2011). For instance, Tanaka et al. (2017) have shown the key role of sodium Nav1.7 channels in several pathological pain syndromes.

The current report provides novel information concerning not only fundamental molecular properties but also strategic topography of neuro-immune crosstalk underlying purinergic and serotonergic signaling and their impact on voltage-gated channels that may contribute to the peripheral mechanisms of migraine pain.

## Materials and Methods

### Model of Meningeal Nociception

**Figure 1:**
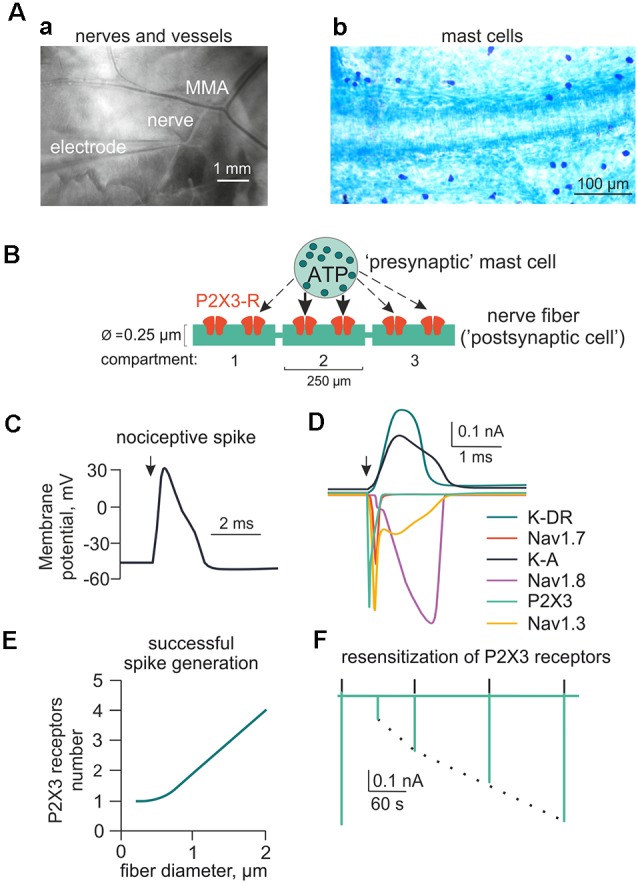
Main components of the model for ATP-induced activation of meningeal afferent (nerve fibers). **(A)** The experimental approach of meningeal spike recording by glass electrode from the local nerve **(Aa)** and the image of meninges with labeled mast cells **(Ab)**. MMA—main meningeal artery. **(B)** Schematic presentation of the model neuroimmune synapse with a mast cell (“presynaptic cell”) as the source of the transmitter of ATP (or 5-HT) and meningeal nerve fiber (“postsynaptic cell”) consisting of several compartments with ATP-gated P2X3 receptors. **(C)** Changes in the membrane potential of the nerve fiber (spike generation) triggered by activation of the P2X3 receptor. **(D)** Ionic currents through several subtypes of sodium, potassium, and P2X3 receptor channels. **(E)** The graph showing the number of P2X3 receptors required to trigger a spike as a function of nerve fiber diameter. **(F)** The kinetics of P2X3 receptor-induced currents and the recovery time course from desensitization.

To simulate rat meningeal trigeminal fiber activity, we used the NEURON environment version 7.5 (Hines and Carnevale, 2003). The fiber was assumed to be 3 cm long (Messlinger, 2009) with a diameter from 0.25 to 2 μm corresponding to C- and Adelta-fibers, respectively. All A-fibers in the dura belong to the Adelta subtype and are known to be present in the meninges (Strassman et al., 2004). Figure 1B shows the basic features of the model with the fiber (green) surrounded by a mast cell containing the secretory vesicles (as sources of eATP or 5-HT; Yegutkin et al., 2016; Kilinc et al., 2017) and forming the neuro-immune synapse (Giniatullin et al., 2019; Koroleva et al., 2019; Nurkhametova et al., 2019). Each segment of the fiber is referred to as a compartment and is indicated as a green rectangle (0.25 μm wide and 250 μm long). In our preliminary tests, we found just one P2X3 receptor in the compartment to be sufficient to depolarize the membrane potential up to the spike threshold (Figure 1D). The properties of the following sodium and potassium channels examined in the present report are listed in Supplementary Table S1: Nav1.7 (Tigerholm et al., 2014), Nav1.8 (Balbi et al., 2017), Nav1.3 (Cummins et al., 2001), potassium delayed rectifier (K-DR; Tigerholm et al., 2014) and A-type currents (Gasparini et al., 2004).

It has been previously shown that meningeal nerves often branch to make a sort of “neuronal dendritic tree” (Schueler et al., 2014). Thus, we simulated a dendritic tree of the trigeminal nerve fiber with two branches. The tree was 3 cm long and comprised 170 compartments: main dendrite (70 compartments), and each branch with 50 compartments (example in Figure 6B). Two branches of the trigeminal nerve were joined at 1.75 cm from the trigeminal ganglion (TG). This is a site that can block the propagation of neuronal signals in the refractory state (Schueler et al., 2014). This property was further explored in simulation experiments. To implement the ATP induced activation of nerve fibers, we integrated the cyclic scheme of the P2X3 receptor operation (Sokolova et al., 2006) into the model. To simulate ATP release from mast cells, we used the 3D diffusion model described by Saftenku (2005) which is based on the following equation:

$$C(x,y,z,t)=2\frac{M}{{\left(4\pi Dt\right)}^{3/2}}\exp(-kt-\frac{x^{2}+y^{2}+z^{2}}{4Dt})$$

where C is the concentration of ATP, D is the diffusion coefficient (0.8 μm2/ms, Helenius et al., 2002), k is the degradation coefficient (Suleymanov et al., 2015), M is the initial amount of ATP released from mast cells, x, y, z are the space coordinates and t stands for time. The ATP profile in the model was simulated as instant rise and slow decay determined by diffusion.

**Figure 2:**
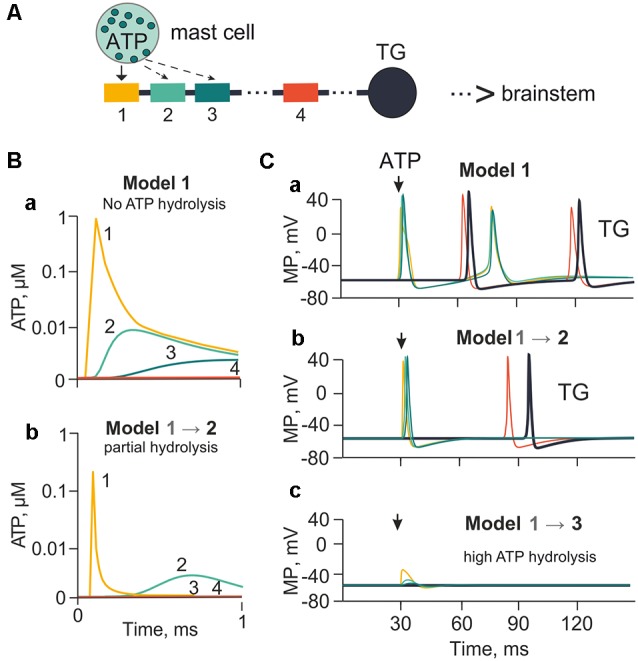
The model of meningeal afferents with different rates of ATP hydrolysis. **(A)** Schematic presentation of the multi-compartment model of neuro-immune synapse with ATP release from mast cells and acting to several neighboring nerve compartments. **(Ba)** ATP concentration *via* diffusion to each (1, 2, 3, 4) nerve compartment without hydrolysis. **(Bb)** ATP concentration at each compartment with partial hydrolysis (degradation coefficient 0.01). **(Ca,b,c)** Spiking activity with no ATP hydrolysis (Model 1), with low ATP hydrolysis (Model 2) and high rate of hydrolysis (degradation coefficient 0.8; Model 3), respectively.

**Figure 3:**
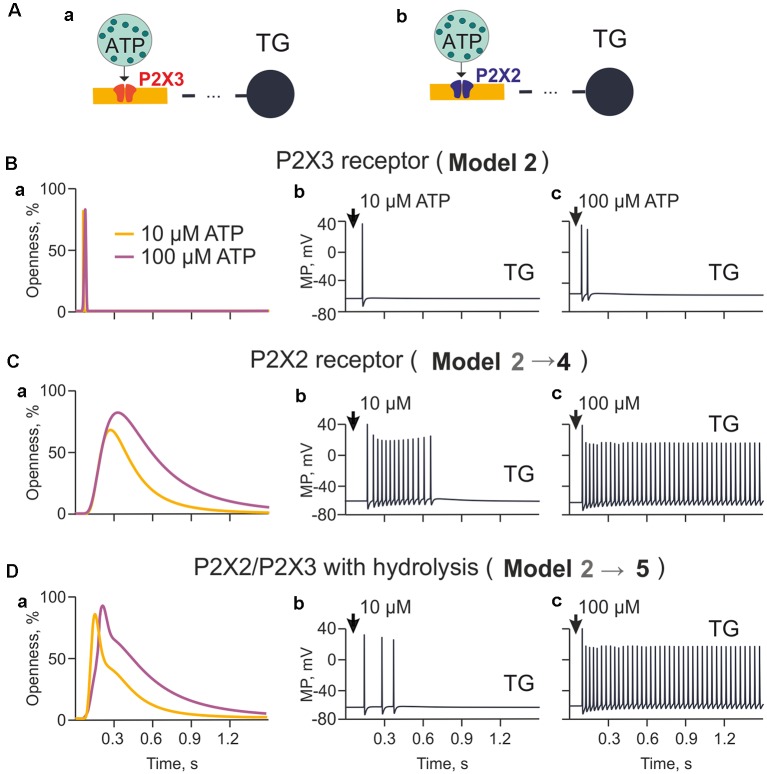
The model of nerve fibers (meningeal afferents) with P2X3 and P2X2 receptors. **(A)** Schematic presentation of the model with P2X3 **(Aa)** or P2X2 receptors **(Ab)**. **(Ba)** The profile of P2X3 receptor responses (openness of receptors) for applications of 10 μM and 100 μM ATP. **(Bb)** Spiking activity induced by activation of P2X3 receptors with 10 μM. **(Bc)** Spiking activity induced by activation of P2X3 receptors with 100 μM. **(Ca)** The profile of P2X2 receptor responses (openness of receptors) for applications of 10 μM and 100 μM ATP. **(Cb)** Spiking activity induced by activation of P2X2 receptors with 10 μM. **(Cc)** Spiking activity induced by activation of P2X2 receptors with 100 μM. **(Da)** The profile of P2X2/3 receptor responses (openness of receptors) with partial hydrolysis for applications of 10 μM and 100 μM ATP. **(Db)** Spiking activity induced by activation of P2X2/3 receptors with 10 μM. **(Dc)** Spiking activity induced by activation of P2X2/3 receptors with 100 μM.

**Figure 4:**
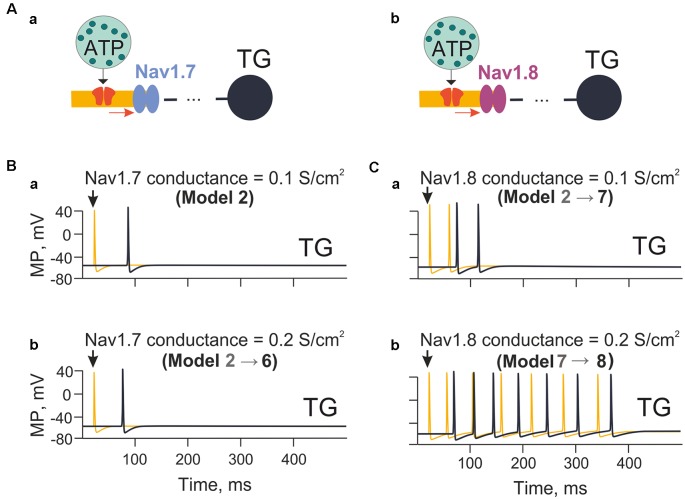
The model with the different contributions of Nav1.7 and Nav1.8 channels. **(Aa)** A model with sodium channels with fast inactivation (Nav1.7). **(Ab)** A model with sodium Nav1.8 channels with slow inactivation. **(Ba)** The spiking activity in the model nerve fiber (Model 2) with Nav1.7 having a conductivity of 0.1 S/cm^2^. **(Bb)** The spiking activity (Model 6) with Nav1.7 having a conductivity 0.2 S/cm^2^. **(Ca)** The spiking activity (Model 7) with Nav1.8 with a conductivity of 0.1 S/cm^2^. **(Cb)** The spiking activity (Model 8) with a conductivity of Nav1.8 equal to 0.2 S/cm^2^. Spiking activity is shown either to the first nerve fiber compartment within the neuroimmune synapse (yellow) or in the trigeminal ganglion (TG; black).

**Figure 5:**
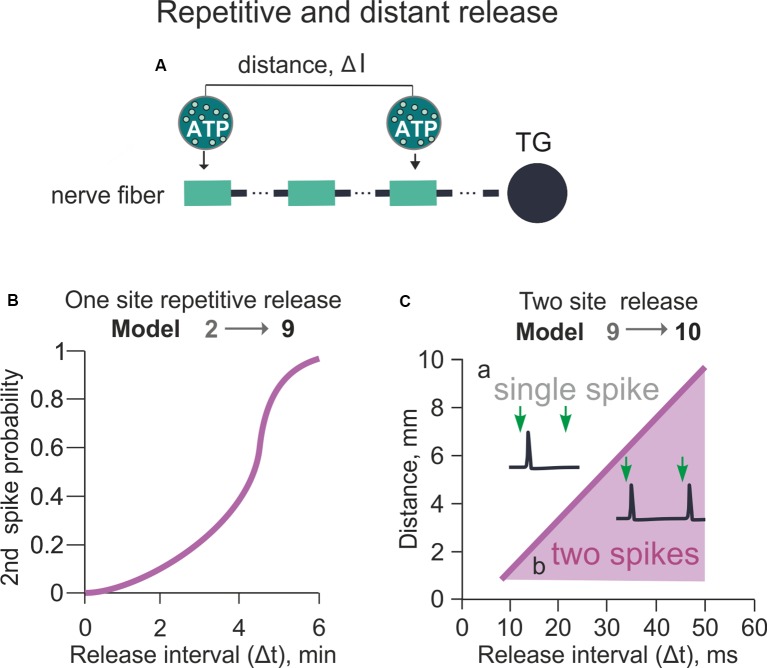
Model of the spiking activity with the repetitive or distant release of ATP from mast cells (variable Δt and Δl between two ATP release events). **(A)** The schematic presentation of the multi-compartment model of a nerve fiber with various distances (Δl) between two ATP applications. **(B)** The probability of successful 2^nd^ spike generation with a variable time (Δt) between two ATP applications. The distance (Δl) between two ATP applications is 0 (Model 9). **(C)** The dependency between the distance (Δl) and time (Δt) between two ATP applications (Model 10). The purple area identifies the conditions for the productive second spike generation (see inset) after two ATP applications whereas white area above—conditions when even a double release of ATP generates only one spike (see inset).

**Figure 6:**
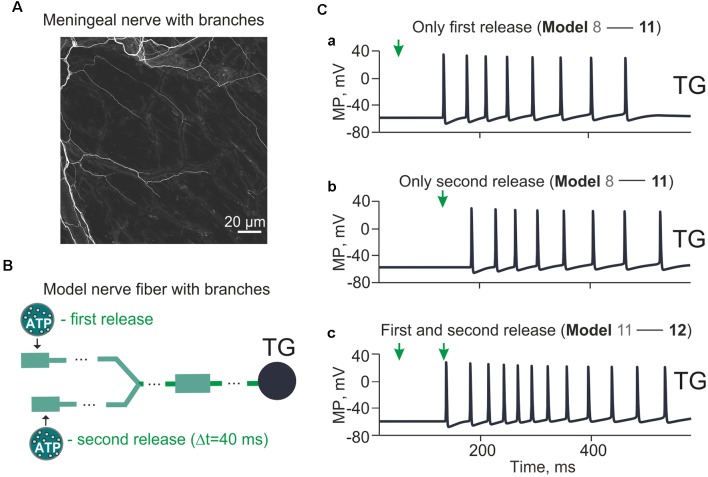
Model with branching meningeal afferents. The spiking activity arriving at the TG from a two-branch structure with Δ*t* = 40 ms between two ATP applications and the distance (Δl) between them 1 cm. **(A)** Image of the nerve fiber with branches. Sections of mouse meninges were stained with the anti-beta-III-tubulin antibody. Single nerves emerging from diffuse bundles of trigeminal fibers are visible throughout the entire section. Bifurcating nerve fibers appear to project directly to meningeal blood vessels (visible from the background staining). **(B)** The “fork” model of the nerve fiber with two separate branches. **(Ca)** The spiking activity induced by a single ATP application at the *t* = 50 ms (Model 11). **(Cb)** The same after a single ATP application at the *t* = 90 ms. **(Cc)** The spiking activity with two ATP applications with Δ*t* = 40 ms (Model 12).

### Experimental Part

The experimental part was performed on 10–12-week-old male WT C57BL/6J mice and adult Wistar rats provided by the Animal Facilities of the University of Eastern Finland (UEF). All procedures were approved by the Committee for the Welfare of Laboratory Animals of the University of Eastern Finland and the Provincial Government of Kuopio. Experiments were conducted according to the European Community Council guidelines (Directives 86/609/EEC). All efforts were made to minimize the number of animals used and their suffering.

### Recording of Spikes From Meningeal Afferents

For the aim of validation, model data were compared with experimental results obtained with application of ATP or 5-HT onto meningeal afferents and published by us earlier (Koroleva et al., 2019). For those experiments we used isolated whole-mount mouse hemiskulls as previously described (Zakharov et al., 2015). Hemiskulls were isolated, keeping the dura mater with meningeal nerves and vessels intact. The meningeal branch of the trigeminal nerve was cleaned from surrounding tissue, then cut and placed inside a saline-filled glass electrode. All recordings of electrical activity from trigeminal nerves were performed from hemiskull preparations continuously perfused with ACSF oxygenated with 95% O_2_/5% CO_2_. Trigeminal nerve spiking activity was recorded using DAM80 amplifier (World Precision Instruments, Sarasota, FL, USA). Electrical signals were digitized using a NI PCI6221 board (National Instruments, USA) and stored on a PC for off-line analysis. Signals were visualized by WinEDR v.3.2.7 software (University of Strathclyde, Glasgow, UK) and analyzed with Matlab-based software (Zakharov et al., 2015).

### Mast Cells Labeling

To demonstrate abundance and localization of mast cells in the meninges we used rat hemiskull preparations as described previously (Shelukhina et al., 2017). After decapitation the head was cut mid-sagittally, brain was carefully removed to leave dura mater untouched. Then preparation was fixed in 4% PFA for 4 h, meninges were mounted on microscopy slides and stained in 0.1% Toluidine Blue.

### Immunolabeling

For nerve fibers staining (tubilin-beta III positive filaments), dissected meninges (from five *ad hoc* prepared mice) were incubated in a blocking solution containing 2% normal goat serum, 1% BSA, 0, 05% Tween20 and 0.1% Triton X-100 (Sigma Aldrich) for 1 h at room temperature. Meninges were incubated overnight at 4°C, with rabbit anti-tubilin-beta III (1:1,000; Sigma T2200) primary antibody. Meninges were then washed three times in PBS and incubated for 2 h in the dark at room temperature with goat anti-rabbit AF488 (Invitrogen A11008) secondary antibody. After washing three times in PBS, meninges were mounted with Fluoromount-G (Thermo Fisher Scientific 00-4958-02), and images acquired with a Zeiss LSM 710 confocal microscope.

## Results

### P2X3 Receptor Probability of Spike Generation

Figure 1Aa shows the two principal components of the meningeal trigeminal system, namely the middle meningeal artery (MMA) and nearby trigeminal nerves with the glass electrode for spike recordings. In Figure 1Ab note the abundance of mast cells located in close proximity to meningeal blood vessels, which is a region densely innervated with trigeminal afferents. For modeling the meningeal neuro-immune synapse, we first focused on the pro-nociceptive action of extracellular ATP (eATP; Burnstock, 1981; Yegutkin et al., 2016) and well-known kinetic properties of eATP-gated P2X2 and P2X3 receptors whose kinetics were presented in the earlier models (Skorinkin et al., 2003; Sokolova et al., 2006).

We assumed that meningeal mast cells known to be in close contact with nerve terminals (Figure 1B) serve as the main source of the local ATP release (“presynaptic cell”). However, we cannot exclude other sources of local eATP as it is well-known that various cell types can release ATP upon mechanical stress (Bodin and Burnstock, 2001; Sperlágh et al., 2007). Among them, there is release of ATP from endothelial cells during shear stress (John and Barakat, 2001; Boileau et al., 2013). As a first approximation, we did not take into account cellular stress as source of ATP release and modeled just eATP diffusion over time. Segments of the nerve fiber (“postsynaptic cell” in the frame of the hypothesis of neuro-immune synapse) in our first models, express ATP-gated P2X3 receptors (Figure 1B). Figure 1C shows a simulated nociceptive spike, while Figure 1D shows sodium, potassium and P2X3 receptor currents underlying this spike. Figure 1E depicts a plot obtained from data modeling to determine the number of receptors necessary for spike generation by a single 250 μm long nerve fiber. We assumed 0.25–2 μm diameter range and observed quadratic dependency of the number of P2X3 receptors for spike generation in one compartment of the nerve fiber. Thus, for the 0.25 × 250 μm compartment, an active P2X3 receptor already effectively generated nociceptive firing. As mentioned earlier, P2X3 receptors possess strong desensitization (Giniatullin and Nistri, 2013). Figure 1F shows the model of slow recovery from P2X3 receptor desensitization as the amplitude of the first current response (green deflection) is decreased following closely spaced ATP application and recovers as time lapses. This property was used in subsequent versions of our model (Models 1–14). As low concentration of ATP can produce the inhibitory action on P2X3 receptors *via* high-affinity desensitization (HAD; Sokolova et al., 2006; Khmyz et al., 2008), we also simulated the prolonged action of 1 nM ATP on the stimulatory effect of this purinergic agonist (Supplementary Figure S2A). First, we found that, indeed, HAD reduced the probability of spikes generation by low concentrations of ATP. Thus, after HAD, the minimal number of active P2X3 receptors required for spike generation was doubled comparing with model without HAD (Supplementary Figure S2Ba). Interestingly, in this test, also the dependance between the number of receptors and the nerve fiber diameter became much steeper. Further, HAD reduced, by half, the amplitude of the P2X3 receptor mediated current induced by 1 μM ATP (Supplementary Figure S2Bb). Finally, the probability of repetitive firing *via* P2X3 receptors with HAD was also reduced as now the release of ATP triggered only one spike (Supplementary Figure S2Cb), whereas without HAD the same release produced two spikes (Supplementary Figure S2Ca).

Because of the large number of tested conditions, Supplementary Figure S1 shows the hierarchy of models used (see also Supplementary Table S3), starting from the simplest model 1 with ATP degradation coefficient = 0, conductance of Nav1.7 = 0.1 S/cm^2^ (Tigerholm et al., 2014) and P2X3 receptor function for the receptor potential necessary for spike generation. In model 2, we set the degradation coefficient of ATP to 0.01. Model 3 originates from model 1 with the degradation coefficient of ATP equal to 0.8. Model 5 stemmed from model 2 with the presence of P2X3 and P2X2 receptors in a 50/50 ratio, whereas in model 4 we used only P2X2 receptors (instead of P2X3 receptors) to obtain activation of the nerve fiber. Model 6 was developed from model 2 with a higher Nav1.7 conductivity (0.2 S/cm^2^) used also for model 7 with Nav1.7 0.1 S/cm^2^ and Nav1.8 0.1 S/cm^2^ conductivity, while model 8 with Nav1.8 0.2 S/cm^2^ conductivity. The subtype Nav1.3 (Cummins et al., 2001) was present in all models with 0.2 S/cm^2^ conductivity. Model 11 is based on model 8 with the fiber topology expressed as a tree, and model 12 was based on the same topology with two ATP release sites.

### The Role of Diffusion and ATP Hydrolysis

It is generally accepted that three ATP molecules must occupy three binding sites to activate successfully a single P2X3 receptor (Sokolova et al., 2006). However, in the tissues related to generation of initial migraine pain, such as the meninges, ATP is rapidly degraded by extracellular NTPDases to the less active ADP, and then to AMP and adenosine (Yegutkin et al., 2016). Hence, in our model we introduced the role of ATP diffusion and hydrolysis in the control of trigeminal afferent firing. First, we explored the action of 1 μM ATP, which is close to its EC_50_ on the P2X3 receptor (Sokolova et al., 2006; Supplementary Table S2). In this simulation we assumed that P2X3 receptors were homogeneously distributed along the nerve fiber. The ATP concentration profile reaching compartments 1, 2, 3 and 4 of the nerve fiber without hydrolysis (lack of NTPDases) or with partial hydrolysis (active NTPDases) is indicated in Figure 2A (compartments 1, 2, 3 were close to the point of ATP release). Compartment 1 was located opposite the release point, whereas the distant compartment 4 was 55 compartments away from the compartment 3 (distance equal to 13.7 mm). The compartment 4, unavailable for ATP, served here only to indicate the propagation of spikes along the fiber. In model 1, in the compartment 1 without hydrolysis (degradation coefficient = 0; Figure 2Ba), ATP was as high as 0.9 μM ATP at the nerve fiber. To simulate ATP hydrolysis, we changed the rate of ATP degradation from 0.01 s^−1^ to 0.8 s^−1^. Thus, model 2 (Figure 2Bb) indicates the profile of ATP concentrations in compartments 1, 2, 3 and 4 with partial hydrolysis. In this case, the maximal ATP concentration in the compartment 1 (0.06 μM) was over 10 times less than in model 1, and was enough to activate 10% of P2X3 receptors. Strong rate of hydrolysis decreased the concentration of ATP in the compartments 2, 3 and 4 to almost undetectable values because of the exponential character of ATP diffusion (Equation 1). Without ATP hydrolysis, P2X3 receptor activation triggered spiking (two spikes) in compartment 1, which propagated through the compartments 2, 3 and 4 to reach the TG (Model 1; Figure 2Ca). In the case of strong hydrolysis (degradation coefficient = 0.01 from maximal 1), there was only one spike produced by 1 μM ATP (Model 2; Figure 2Cb). Neuronal activity decreased dramatically when the degradation coefficient was 0.8 (close to the maximal value of 1). In this case, even in compartment 1, the local receptor potential did not reach threshold for spike generation (Model 3; Figure 2Cc).

### Comparing the Role of P2X2 vs. P2X3 Receptors and Effect of P2X2/3 Heteromers

Although P2X3 receptors are expressed in approximately 80% of trigeminal neurons (Simonetti et al., 2006), sensory neurons also express slowly desensitizing P2X2 receptors which are implicated in pain signaling (Lewis et al., 1995; Fields and Burnstock, 2006). Thus, we next explored the differential ability of P2X2 and P2X3 receptors to support spiking activity in trigeminal fibers (Figures 3Aa,b). First, given that cells can release more than 1 μM ATP, we tested the role of higher concentrations of ATP limited by hydrolysis (Model 2; Figure 3Ba) in the firing activity triggered by a homogeneous population of P2X3 receptors. Because of slow desensitization (Sokolova et al., 2006), we found that ATP signaling *via* P2X3 receptor provided just one (10 μM ATP) or two (100 μM ATP) spikes propagating to the TG (Figures 3Bb,c, respectively).

Next, we simulated the role of P2X2 receptors based on a kinetic model with minimal desensitization state, which is a typical feature of this receptor (Skorinkin et al., 2003; Moffatt and Hume, 2007). Unlike P2X3 receptors, this approach (Model 4) yielded much more prolonged receptor activation (Figure 3Ca) and multiple firing in the TG especially in the case of 100 μM ATP (Figures 3Cb,c).

Because the potential co-expression of P2X2 and P2X3 receptors with distinct desensitization rates in trigeminal neurons (Simonetti et al., 2006) and because P2X2 receptor subunits can co-assemble with P2X3 ones to form P2X2/P2X3 heteromers (Fields and Burnstock, 2006), we also explored the effect of such co-assembly (comprising 50% P2X3 and 50% P2X2) on meningeal neuronal firing (Model 5). Figure 3Da indicated that in such a case, despite hydrolysis, 10 μM ATP produced repeated spikes (Figure 3Db), although less intensively than in the case of homogeneous P2X2 receptors (Figure 3Cb). Simulating the release of 100 μM ATP led to strong, prolonged TG spiking (Model 5; Figure 3Dc). Thus, co-expression of P2X2 and P2X3 receptors appeared to be a powerful process to generate sustained nociceptive activity.

### Different Role of Nav1.7 and Nav1.8 Sodium Channels

Among the subtypes of sodium channel expressed by nociceptive neurons, the subtypes Nav1.7 and Nav1.8 play a special role in the onset and propagation of spiking activity (Tigerholm et al., 2014; Balbi et al., 2017). Thus, we explored the impact of Nav1.7 and Nav1.8 channels on ATP-induced firing of trigeminal fibers (Figures 4Aa,b). First, we tested the role of Nav1.7 subunit density on fiber activity induced by a single ATP release event (ATP 1 μM) acting on P2X3 receptors in compartment 1 (Model 2; Figure 4Ba). When the Nav1.7 conductance was 0.1 S/cm^2^, and the P2X3 receptor current depolarized membrane potential to −20 mV, this condition was sufficient to activate sodium channels and trigger limited spiking (Figure 4Ba). When the Nav1.7 conductance was doubled to 0.2 S/cm^2^, the propagation rate of spikes was slightly increased, yet the number of spikes was unchanged (Figure 4Bb).

Similar tests were performed with Nav1.8 channels (co-expressed with Nav1.7) starting from 0.1 S/cm^2^ conductance like with Nav1.7 channels. In this case, after a single ATP release event (ATP 1 μM), multiple spiking emerged (Figure 4Ca). In contrast to Nav1.7, firing largely increased when the conductance of Nav1.8 channels was doubled (Figure 4Cb). During these simulations the activity of potassium channels was not changed, implying that changes in certain sodium channels (especially Nav1.8 subtype as indicated in models 7 and 8) were already sufficient to dramatically affect spiking activity of the nerve fiber.

### Repetitive ATP Release and Distant Release Sites Along the Nerve Fiber

Because meninges contain an abundance of mast cells along the vessels and the divergent branches of the sensory fibers (Theoharides et al., 2007; Figure 1Ab), we next explored the role of repeated ATP release on the probability of repetitive spike generation in this histological arrangement. First, we simulated two release events delivering ATP to same site of the fiber (Model 9). This model consisted of 120 compartments along the nerve fiber, each compartment equipped with Nav1.7, Nav1.3, potassium DR, and potassium A-type conductances. In order to identify factors that can overcome lingering P2X3 desensitization, we again used signaling only *via* P2X3 receptors. This approach provided an initial condition with one ATP-driven spike to facilitate detection of other factors leading to multiple firing.

Using model 9, we set the different timing (Δt) of the two ATP release events in a range of 2–8 min and investigated generation and propagation of spikes from the release point to the TG. As expected, the generation of a second spike in the basal model with a simple one-release point simulation was mostly determined by the slow recovery of P2X3 receptors from desensitization. Thus, we did not observe a second spike with two ATP applications separated by less than 4 min Δt interval. Hence, spikes were generated only when the second release occurred 6 min after the first one (Figure 5B). The probability of the second spike firing was nonlinearly dependent on Δt (Figure 5B). Thus, at *Δt* = 5 min the generation of a second spike had 0.5 probability, whereas at Δt ≥ 6 min the function reached saturation with probability 1 for the second spike generation.

We next focused on the effect of the distance (Δl) between two distinct ATP release points (spatial factor) located along the nerve fiber to evoke a second spike and overcome the long-lasting desensitization of P2X3 receptors (Figure 5A). The minimal starting distance between two release sites was set at 1 mm to minimize the effect of ATP diffusion from the first release site (Figure 5B). Then, we varied the distance (l) and time (t) between two ATP applications (Model 10; Figure 5C). Figure 5Ca indicates that at *Δt* = 20 ms and *Δl* = 5 mm, only one spike emerged despite a dual ATP release, whereas at *Δt* = 40 ms and *Δl* = 7 mm two spikes appeared in the same fiber upon the second ATP release (Figure 5Cb). Figure 5C shows the relation between Δt and Δl for generation of the second spike after two ATP release events. Thus, for triggering more than one spike, the minimal Δt value was 15 ms with 1 mm Δl distance between two ATP release events (Figure 5C).

In summary, in the present simplified model with the linear morphology of the nerve fiber and only two mast cells as source of ATP, the probability of the second spike generation was nonlinearly dependent on the timing of the second ATP application (Δt) and was linearly dependent on the distance between ATP release events (Δl).

### Complex Architecture of Trigeminal Fibers

We further developed our model to include a simple bifurcation of the single nerve fiber since it has been shown significant branching of trigeminal fibers in meninges (Schueler et al., 2014). The morphology of mouse meningeal innervation with the branched nerve fibers is shown in Figure 6A. The schematic presentation of the branched tree is presented in Figure 6B. Model 11 shown in Figure 6B consists of 170 compartments in two branches, each one with a P2X3 receptor and Nav1.8, Nav1.7, Nav1.3, potassium DR and A-type conductances. The bifurcation point was 70 compartments (1.75 cm) away from the TG, and there were two ATP release points 1 cm apart.

First, we found that a single (first) ATP release (Figure 6C, indicated by arrow at simulation time = 50 ms) triggered (after some latency) multiple spiking of the TG branch (eight spikes in the period from 152 up to 485 ms; Figure 6Ca). Second, we tested the impact of a second ATP release (40 ms later than the first one) that also triggered repetitive spiking in the other branch of the nerve (eight spikes, activity lasting from 193 ms to 538 ms; Figure 6Cb). Then, we explored the firing activity of a whole nerve sequentially activated by ATP released from two (first and second) distinct sites. This resulted in stronger spiking (12 spikes, activity lasting from 152 ms to 538 ms; Figure 6Cc) which reached the TG.

Thus, the complex architecture of trigeminal fibers can also contribute to multiple firing.

### 5-HT Induced Activation of Nerve Fibers

**Figure 7:**
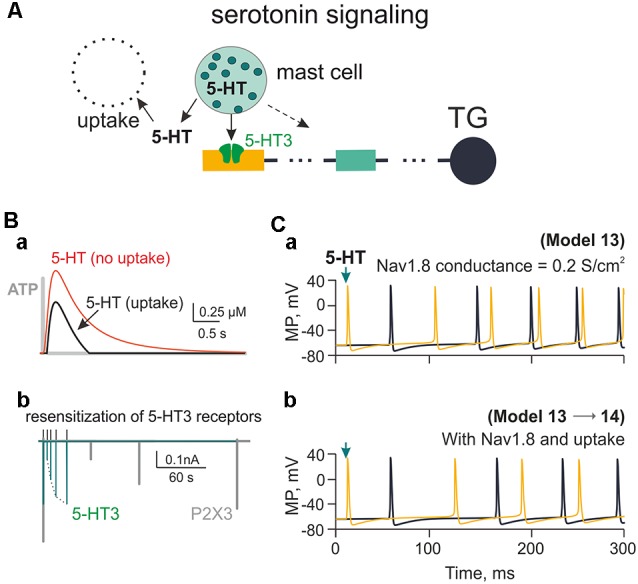
Firing of meningeal afferents induced by 5-HT applications. **(A)** The multi-compartment model of the nerve fiber with 5-HT3 receptors activated by 5-HT released from mast cells and inactivated through uptake mechanism. **(Ba)** The red graph is the time profile of the extracellular concentration of 5-HT without uptake (Model 13), whereas the black graph is 5-HT concentration with uptake (Model 14). For the sake of comparison between the two mediators, the gray graph shows the very short profile of ATP concentration (Model 1). **(Bb)** The green graph shows 5-HT3 receptor recovery from desensitization, whereas the gray graph—the comparative slow desensitization of P2X3 receptor. **(Ca)** The spiking activity of the nerve fiber activated by 5-HT with Nav1.8 having the conductivity of 0.2 S/cm^2^ (Model 13). **(Cb)** The same with 5-HT re-uptake (Model 14). Spiking activity is shown either to the first nerve fiber compartment within the neuroimmune synapse (yellow) or in the TG (black).

Mast cells containing the principle mediator 5-HT are largely expressed in the meninges (Figure 1Ab). We have previously shown the strong pro-nociceptive action of this monoamine on meningeal afferents *via* ligand gated 5-HT3 receptors in rats (Kilinc et al., 2017) and mice (Koroleva et al., 2019). In the present study, we applied the kinetic model of 5-HT3 receptors (Corradi et al., 2009) to our model of C-fibers. The model implied mast cells releasing 2 μM 5-HT acting on 5-HT3 receptors and subjected to 5-HT uptake (Figure 7A). For the sake of simplicity, we indicated only two compartments of the trigeminal nerve. Figure 7Ba shows the plot of 5-HT concentration with or without 5-HT uptake (Daws et al., 2005; Wood et al., 2014). Unlike the short time profile of ATP (rapidly degraded by ectoenzymes, gray trace), the concentration of 5-HT after single release from mast cells decayed much slower with or without uptake processes (Figure 7Ba). In addition, 5-HT3 receptor recovery from desensitization was also faster (Figure 7Bb; green) than the one for the ATP-gated P2X3 receptor (Figure 7Bb, gray). Comparison of repeated spiking activity triggered by 5-HT3 receptor activation with or without monoamine reuptake and contribution of Nav1.8 channels is shown in Figures 7Ca, Cb (Model 13 and 14). Spiking activity was, however, more intense with ATP application and Nav1.8 channel activity (Figure 4Cb).

### Comparison of Model and Electrophysiological Data

**Figure 8:**
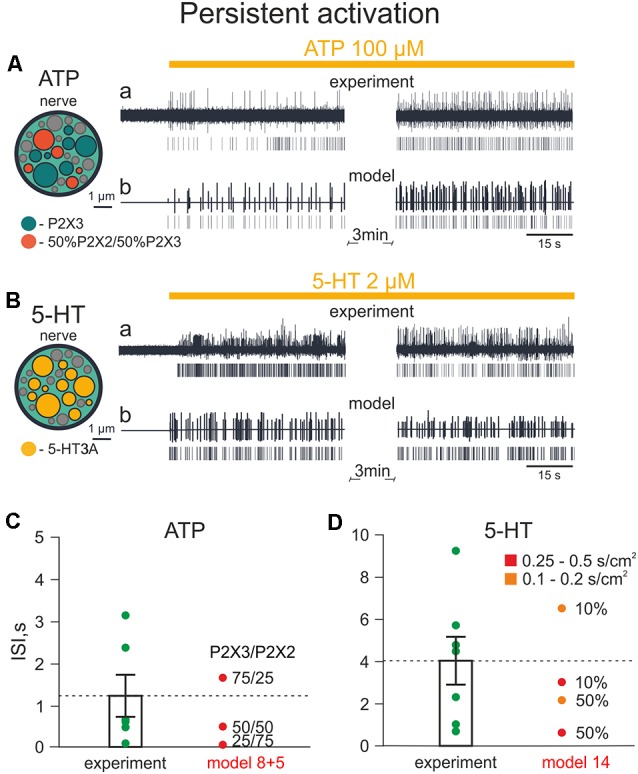
Firing of meningeal afferents induced by *persistent* ATP and 5-HT applications. **(A)** A cross-section of the model nerve with P2X3 receptors and with a mixture of 50% P2X2 + 50% P2X3 receptors. **(Aa)** The spiking activity of fibers with persistent ATP application in a biological experiment. **(Ab)** The same in the model. **(B)** The cross-section of the model nerve with 5 HT3 receptors. **(Ba)** The spiking activity of fibers with persistent 5-HT application in a biological experiment. **(Bb)** The same in the model. **(C)** The inter-spike intervals (ISI) with persistent ATP application. **(D)** The inter-spikes intervals with the persistent 5-HT application.

To validate our model, we compared simulated firing with experimentally observed action potentials triggered by ATP and 5-HT on mice meningeal afferents (Koroleva et al., 2019). In this study, we measured the firing of the mouse trigeminal nerve activated by application of exogenous ATP. Figure 8Aa shows the effect of 100 μM ATP for the 1st and 5th min of its action in meninges.

Next, we compared these experimental results with the simulation of neuronal activity induced by 100 μM ATP (Figure 8Ab). Figure 8A (inset, left) shows a cross section of the model nerve with several active nerve fibers: either with P2X3 receptors or with a mixture of P2X2 and P2X3 receptors (Models 5 and 8, respectively). The inactive fibers (shown in gray) represent the absence of P2X receptors. Like the experimental approach, our model revealed an intense and prolonged ATP-induced spiking activity in the nerve fiber.

Comparable results were obtained also with experimental and simulated applications of 5-HT. Figure 8B shows the structure of the model nerve in which fibers express 5-HT3 receptors or lacking such receptors. In this case, as experimental application of 2 μM 5-HT induced a long-lasting activity in meningeal afferents, a similar phenomenon was also observed with simulated application of 2 μM 5-HT (Model 14; Figures 8Ba,b).

Then, we compared the inter-spike intervals (ISI) of experimental and modeling data (Figures 8C,D). In experimental ATP application, ISI were 1,213 ± 499 ms, whereas in the model, the median ISI were 502 ms with 50%/50% mixture of P2X2 and P2X3 receptors, 1,704 ms with 75%/25% mixture and 56 ms with 25%/75% mixture (Figure 8C). Thus, the 75%/25% ratio of P2X3/P2X2 receptors in the model was the closest combination of P2X2 and P2X3 receptors to the experimental data.

With experimental 5-HT application, the ISI of spiking activity was 4,039 ± 1,134 ms, and the closest model data were obtained with 10% activated fibers which had 5-HT3 receptors with conductances ranging from 0.25 to 0.5 S/cm^2^ and 0.1–0.2 S/cm^2^ (ISI: 3,041 ms and 6,556 ms, respectively; Figure 8D).

Together, these data indicated a high similarity of our model results with experimental ATP- and 5-HT-induced firing.

### Modeling Prolonged and Rhythmic Nerve Activity

Migraine pain has a still unexplained pulsating character perhaps due to mechanical fluctuations of meningeal vessels (Mikhailov et al., 2019; Della Pietra et al., 2020) that might interact with nearby nerve fibers and mast cells (Figures 1Ab, 6A). In order to investigate the potential role of pulsating blood flow as a trigger of meningeal nociception and its relation to ATP- and 5-HT-induced firing, we hypothesized that vasodilation (during migraine attacks) of pulsating meningeal vessels can stimulate release of these two mediators from local mast cells. In addition, we considered that ATP can be released directly from endothelial cells of meningeal vessels (Burnstock, 1981).

**Figure 9:**
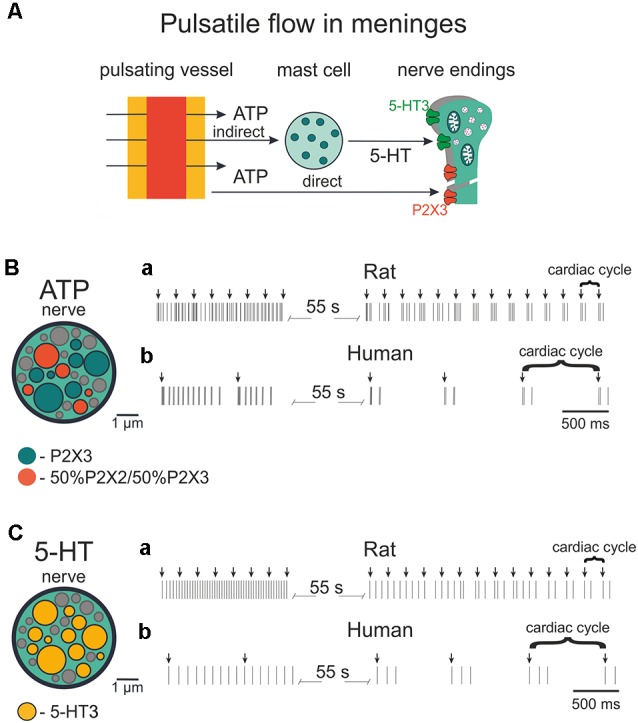
Firing of meningeal afferents induced by *pulsatile* ATP and 5-HT applications.** (A)** The schematic presentation of pulsating vessels inducing transmitter release from mast cells or endothelium. ATP acts either directly on nerve terminals (*via* P2X3 receptors) or by 5-HT released from mast cells and 5HT activates its own neuronal 5-HT3 receptors. **(B)** The cross-section of model nerve with P2X3 receptors and a mixture of 50% P2X2 and 50% P2X3 receptors. **(Ba)** Spiking activity in nerve fibers with ATP release events following at the frequency of the human heart rate (70 beats per minute). **(Bb)** The spiking activity of fibers with ATP action at the frequency of the rat heart rate (400 beats per minute). **(C)** The cross-section of the model nerve with 5-HT3 receptors. **(Ca,Cb)** Spiking activity of nerve fibers with ATP action at the frequency of the human heart rate (70 beats per minute) and rat heart rate (400 beats per minute), respectively.

Based on the co-assembly of meningeal nerves, mast cells and vessels shown in Figures 1Aa,b and on our experimental data obtained from mice lacking mast cells (Koroleva et al., 2019), we hypothesized (Figure 9A, shown by arrows) that the pulsatile blood flow can release ATP and 5-HT from mast cells adding more ATP released from the endothelium of meningeal vessels. The extracellular ATP (eATP) can act either directly on nerve fibers or indirectly *via* degranulation of mast cells. These interactions are also shown in the (Supplementary Movie).

The structure of modeled nerves for ATP and 5-HT actions in Figures 9B,C (insets, left) was the same as shown in Figure 8. First, we modeled the neuronal activity in the rat induced by periodic pulsations (assuming an average heart beating rate = 400/min; Figures 9Ba, Ca). Thus, we examined the spiking activity assuming that these pulsations released 1 μM eATP (note arrows in Figure 9Ba) which, in turn, activated nerve fibers. Interestingly, after the initial continuous spiking induced by ATP, the neuronal discharges (indicated by short vertical lines in Figure 9Ba) occurred in short bursts separated by longer silent periods, thus demonstrating a clear periodicity.

Modeling also allows simulating pulse-induced spiking in human afferents. Figure 9Bb for ATP and Figure 9Cb for 5-HT, show that the assuming human pulsating blood flow (presumed to be 70 beats/min) could produce initial persistent firing, which was quickly transformed into periodic bursts of activity with expected lower rate. In this case, we took into consideration not only the lower human-specific heart rate but also the faster (*circa* two-fold) rate of recovery from desensitization of human P2X3 receptors (Pratt et al., 2005).

## Discussion

The main result of the current study is a novel model of meningeal nociception based on the concept of a neuro-immune synapse composed by trigeminal nerve fibers and local mast cells, which may release two mediators, ATP and 5-HT. These two endogenous substances were taken here as chemical triggers of nociception based on their powerful and prolonged stimulatory effect on meningeal afferents experimentally demonstrated with direct recordings of spikes from trigeminal nerve fibers in rats and mice (Yegutkin et al., 2016; Kilinc et al., 2017; Koroleva et al., 2019).

### Role of ATP Receptor Subtypes

To model purinergic (eATP-driven) signaling within the neuro-immune synapse, we first simulated the kinetic properties of eATP-gated P2X2 and P2X3 receptors, which are the major ATP sensitive receptor subtypes in sensory neurons generating pain signals (Burnstock, 2006; Simonetti et al., 2006).

Because of the fast onset of desensitization and slow recovery properties (Sokolova et al., 2006), P2X3 receptors activated by a single pulse of eATP (even without any ATP hydrolysis) evoked only limited activity in meningeal trigeminal nerve fibers (Model 1; Figure 2Ca). Assuming moderate ATP hydrolysis to mimic short ATP lifetime in meninges (Yegutkin et al., 2016), repetitive firing of trigeminal fibers was transformed just into a single ATP-driven spike (Model 2; Figure 2Cb). Robust ATP hydrolysis left only a small receptor potential unable to trigger nociceptive spikes (Model 3; Figure 2Cc).

One crucial factor for the intensity and persistence of firing was the ATP receptor subtype. Unlike P2X3 receptors widely (up to 80%) expressed in the rodent trigeminal neurons (Simonetti et al., 2006), P2X2 receptors are less frequently expressed by sensory neurons, yet they show little desensitization despite relatively fast activation properties (North, 2004). Thus, the current simulations assuming a single release point indicate that the slow desensitization kinetics of P2X2 receptors could ensure high probability of repetitive firing (Model 4; Figure 3C).

The relative prevalence of P2X2 and P2X3 receptor subtypes is species and sensory neuron dependent (Simonetti et al., 2006; Viatchenko-Karpinski et al., 2016; Ishchenko et al., 2017). In the human DRG, there is a predominance of the P2X3 subtype (Serrano et al., 2012), whereas in rodents, the P2X2 subtype is significantly present in trigeminal neurons (Simonetti et al., 2006). It should be noted that P2X2 and P2X3 subunits can heteromerize to form P2X2/3 heterotrimers (Burnstock, 2000). It has been proposed that P2X3 homomers are responsible for acute pain, whereas P2X2/3 heterotrimers are involved in chronic pain (Jarvis, 2003). Consistent with this view, our simulations indicated that the co-expression of P2X2/P2X3 receptors supported prolonged firing of fibers activated by eATP (Model 5; Figure 3D). Interestingly, unlike homomeric P2X3 or P2X2 receptors, in the case of heteromers, we observed strong increase in neuronal firing after raising the concentration of ATP from 10 μM to 100 μM (Model 5; Figures 3Db,c). Thus, co-expression of P2X2 and P2X3 subunits enabled robust firing sensitive to ATP concentration.

The issue of relative contribution of parallel co-expression in the same neurons of homomeric P2X3 and homomeric P2X2 or the co-assembly of P2X2 and P2X3 subunits in the P2X2/3 heteromers is not fully solved in experimental setting. However, the nociceptive action of the P2X3 agonist α,β-meATP in rat meninges was comparable with the action of ATP (Yegutkin et al., 2016), suggesting that the essential fraction of ionotropic ATP receptors in nerve terminals is presented by P2X2/3 heteromers. This issue might have a significant impact for suitability of P2X3 and P2X2/3 inhibitors for treatment of migraine pain. For instance, potent P2X3 and P2X2/3 inhibitors are already under the advanced stage of clinical trials for the treatment of chronic coup (Richards et al., 2019). On the other hand, highly potent P2X3 inhibitors such as various peptide toxins have been reported (Lalo et al., 2001; Grishin et al., 2010). Even emerging migraine treatments such as cannabinoids (Leimuranta et al., 2018) directly inhibited P2X2 and P2X2/3 receptors in sensory neurons (Krishtal et al., 2006) which can provide a dual anti-nociceptive effect. As ATP in living tissues is quickly degrades to ADP, it is also interesting to consider potential action of ADP *via* P2Y receptors, which might be the additional factor shaping the purinergic nociception in meninges. ADP sensitive excitatory receptors are expressed both in trigeminal neurons and in glial cells (Villa et al., 2010). In contrast, the inhibitory action of ADP on P2X3 receptors has been shown in isolated DRG neurons (Gerevich et al., 2007). However, our experimental study (Yegutkin et al., 2016) did not show significant changes in the nociceptive firing of meningeal afferents after application of ADP suggesting that this type of modulation preferential takes places at the level of neuronal somata in the trigeminal ganglia or DRG. We cannot exclude also the region-specific differences in the relative contribution of P2Y vs. P2X receptors in analogy to the condition detected in the colon where the role of ADP sensitive P2Y receptors is dominating (Hockley et al., 2016).

According to the purinergic hypothesis of migraine (Burnstock, 1981), the powerful algogen eATP is one of key mediators of this disease. In accordance with this notion, we have recently found that, when applied to mouse meninges, 100 μM ATP causes very strong firing (~25-fold increase) of primary afferents (Koroleva et al., 2019). Our modeling of purinergic mechanisms at the meningeal neuro-immune synapse was facilitated by the known kinetics of P2X3 and P2X2 receptors (Skorinkin et al., 2003; Sokolova et al., 2006). The availability of these input model parameters allowed exploring various factors determining the pattern of nociceptive fiber activity when mast cells were assumed as the source of ATP release. Nonetheless, one should bear in mind that, apart from mast cells, meninges are enriched with other immune cells (McIlvried et al., 2017). Thus, in the natural environment of these tissues, there could be multiple ATP releasing cell types possibly contributing in concert to neuro-immune interactions.

### Nociceptive Signaling in Meningeal Afferents *via* 5-HT

Along with eATP, we simulated the action of 5-HT, which is a classical mediator released from mast cells. Recently we showed that 5-HT has a powerful pro-nociceptive action on rat and mouse meningeal afferents mainly *via* ligand-gated 5-HT3 receptors (Kilinc et al., 2017; Koroleva et al., 2019). The selection of 5-HT among other candidate transmitters was further supported by our experimental finding that histamine, also the known as the mast cell mediator, has only little if any excitatory action on nerve terminals (Kilinc et al., 2017). Unlike ATP, the lifespan of extracellular 5-HT in the meninges is expected to be much longer due to relatively slow reuptake (Daws et al., 2005; Wood et al., 2014). Furthermore, 5-HT3 receptors recover from desensitization much more rapidly than P2X3 receptors (Figure 7Bb), rendering 5-HT potentially effective to trigger nociception in meninges. Nevertheless, ATP P2X3 receptors possess higher affinity (EC50 1 μM; Sokolova et al., 2006) and widespread expression in the majority of trigeminal neurons (Simonetti et al., 2006). Interestingly, at other non-traditional “synapses,” such as taste buds, release of ATP and 5-HT also activates nearby nerve terminals *via* P2X2/3 and 5-HT3 receptors (Larson et al., 2015), indicating that these two transmitters and their receptors may operate in a broader context than the proposed meningeal neuro-immune synapse. It is suggested that the functional outcome of dual activation *via* P2X and 5-HT3 receptors of afferents can multiply the total firing in meningeal nociception as a putative signal of migraine pain. The leading role in such scenario, most likely, belongs to ATP, which, apart from the direct excitation of terminals *via* P2X receptors, can degranulate meningeal mast cells to release serotonin, acting *via* excitatory 5-HT3 receptors (Koroleva et al., 2019).

This novel dual purinergic/serotonergic signaling reveals a new translational aspect of the present study, suggesting pharmacological interventions based on a combination of P2X3 and 5-HT3 antagonism.

### Role of Sodium Channels

For effective traffic of nociceptive signals to higher pain centers, sensory neurons must be equipped with a palette of sodium channels, which determine the fidelity and precision of neuronal sensory coding (Zhao et al., 2016). Several subtypes of sodium channel, including Nav1.7, Nav1.8 and Nav1.9 isoforms, are expressed in the peripheral nervous system (Israel et al., 2019). Both the Nav1.7 and Nav1.8 subtypes found in nociceptive neurons can generate depolarizing slow currents with the characteristics to interact with the receptor potential evoked by ATP and 5-HT. In particular, human Nav1.8 channels display slower inactivation kinetics and produce large persistent currents than those observed in other species (Han et al., 2015). In the present model we used the same set of sodium channel data reported by Mandge and Manchanda (2018), and we observed that slow inactivation of Nav1.8 channels is one important determinant of the long-lasting pattern of spiking. Thus, unlike Nav1.7 channels, simulation with high density of Nav1.8 expression largely increased firing activated by ATP *via* P2X3 receptors (Figure 4). Thus, Nav1.8 channels could supply an additional mechanism to amplify the initial activation of nerve fibers by P2X3 receptors.

### Modeling Multiple Activation Sites

Our modeling with a simple linear structure of the nerve fiber indicated that non-desensitizing P2X2 receptors and Nav1.8 channels could efficiently promote persistent firing after P2X3 receptor activation. We further explored other factors to transform the brief firing evoked by P2X3 receptors into a repetitive discharge. Emphasis on P2X3 receptors as initial condition was based on their wide expression by trigeminal sensory neurons (Simonetti et al., 2006), lack of inflammatory pain in mice genetically ablated for this receptor (Cockayne et al., 2000) and by poor expression of P2X2 subunits by human nociceptive neurons (Serrano et al., 2012).

To this end, we sought to improve our model by approximating its structure to the branching of the nerve fibers recently documented by functional and morphological data in dural afferents (Schueler et al., 2014). One unexpected result was that P2X3 receptors alone were sufficient for repetitive firing when ATP was supposed to act on distinct branches of the trigeminal nerve (Model 12). In Model 12, we also quantified the spatial and temporal requirements, which determined the appearance of the second spike. Thus, our study showed that fiber branching could play a major role to generate repeated firing despite the intrinsic desensitization properties of the P2X3 receptor. Simulations indicated that this phenomenon could occur when ATP was applied to distinct branches with at least 40 ms interval. It seems likely that, in the naturally more complex structure of meningeal nerves and multiple release sites, repeated firing of trigeminal fibers equipped solely with P2X3 receptors might be even stronger as desensitization of P2X3 receptor represents a local phenomenon maintaining distant sites of the axonal tree sensitive to ATP.

Focusing on the factors promoting repetitive firing we also should consider factors which potentially can prevent it. Thus, the use-dependent inhibition of P2X3 receptor by low nanomolar concentrations of ATP known as HAD (Sokolova et al., 2006; Khmyz et al., 2008) can reduce the operations of ATP *via* P2X3 (but not *via* P2X2) receptors. In our model we confirmed this possibility showing that HAD can reduce but not abolish the activity of homomeric P2X3 receptors. However, HAD was described in isolated neurons and was not previously tested in *ex vivo* preparations like our model of meningeal nociception and it is unknown whether it takes place *in vivo*. Thus, the (patho)physiological significance of HAD remains unclear and its efficiency *in vivo* could be reduced or neutralized by activity of ATP degrading NTPDases expressed in meningeal afferents.

Thus, the present study suggests how, despite their strong desensitization, P2X3 receptors can contribute to nociceptive pain signaling especially when supported by co-expression of P2X2 receptors, Nav1.8 sodium channels, co-release of 5-HT and branching of nerve fibers.

**Figure 10:**
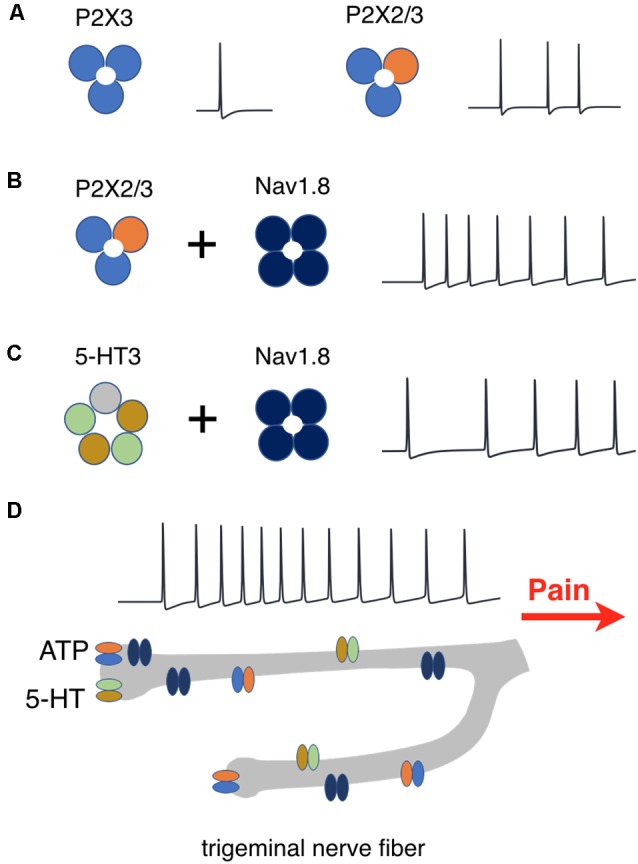
Schematic presentation of factors determining repetitive activation of meningeal afferents.** (A)** P2X3 receptors activation produces one spike whereas activation of P2X2/3 heteromers triggers prolonged activity. **(B)** The co-expression of P2X2/3 or **(C)** 5HT-3 receptors with Nav1.8 channels enhances prolonged spiking activity. **(D)** Nerve branching further increases the duration of spiking activity which signals pain to higher brain centers.

These data are schematically summarized in Figure 10, where the efficiency of repetitive firing *via* only P2X3 vs. co-assembly of P2X3 with P2X2 (Figure 10A), effect of Nav1.8 for P2X2/3 (Figure 10B) and for 5-HT3 receptors (Figure 10C) are indicated. Figure 10D showed the amplifying firing effect of neuronal branching along with other factors.

These novel results provide the clue to solve the paradox how strong P2X3 desensitization is consistent with *in vivo* evidence on the role of P2X3 in pain behavior (Cockayne et al., 2000; North, 2004).

### Potential Pathophysiological Implications in Migraine

Our modeling approach together with previously published data provides a functional scenario to account how, during migraine attacks, ATP and 5-HT can persistently excite meningeal afferents. We assumed here the ATP and 5-HT co-release by meningeal immune cells (exemplified as mast cells). Mast cells react to mechanical stimuli (Zhang et al., 2012; Komiyama et al., 2014), which can serve as a signal to release the content of their secretory granules including ATP (Wang et al., 2013) to activate neighboring nerve fibers (Wang et al., 2015). Interestingly, cortical spreading depression (which underlies migraine aura, Charles, 2018), also degranulates mast cells and opens pannexin channels as the pathway for ATP release (Karatas et al., 2013). Further studies are necessary to identify all the endogenous triggers for 5-HT and ATP release during a migraine attack, also taking into account the blood flow-dependent endothelial release of ATP (Wang et al., 2016) due to shear stress or blood vessel pulsations (John and Barakat, 2001).

As migraine pain is not only long-lasting but also pulsating (throbbing), one aim of this project was to elucidate the factors which provide such migraine-typical activation of trigeminal fibers. Hence, to simulate the pulsating character of migraine pain, which is one of the main features of migraine [The International Classification of Headache Disorders 3rd edition (ICHD-3)], we compared the effect of experimental application of ATP or 5-HT to meningeal nerve fibers with similar modeling tests. Then, based on Models 5 and 8, which provided the best match with the experimental approach, we reconstructed pulsatile release of the two mediators ATP and 5-HT to monitor the spiking activity of nerve fibers.

Furthermore, we simulated the effect of rhythmic pulse-induced release of ATP and of 5-HT not only in rodents but in human nerves. In all cases after initial continuous activity, there were short very regular bursts of nociceptive activity, specific for each species. Notably, such a type of activity with high frequency of spikes within the burst should increase the probability of temporal summation and the windup phenomenon to transmit peripheral signals to the second order nociceptive neurons located in the brainstem (Zakharov et al., 2016). Although release of chemical mediators by pulsating blood vessels cannot *per se* be the primary process for migraine pain, it is feasible to assume that after the initial stimulation of ATP (and 5-HT) release by certain triggers (whose identity needs further study), pulsatile release might support long-lasting firing. Within this framework, the present basic model of meningeal nociception does not consider the early process of neuronal sensitization. Thus, it has been shown that the main migraine mediator, the neuropeptide CGRP largely increases the activity of P2X3 receptors and reduces their desensitization (Fabbretti et al., 2006; Giniatullin and Nistri, 2013). Notably, mechanical activation of peptidergic nerve fibers containing CGRP can perhaps be achieved through mechanosensitive piezo channels (Mikhailov et al., 2019; Della Pietra et al., 2020; and see Supplementary Movie). This effect plus other sensitization mechanisms should enhance the role of ATP in nociceptive firing of meningeal afferents and supply the trigger for resilient firing. In fact, mast cells, apart from 5-HT, release other active substances such as NGF and histamine (Bonini et al., 2003; Borriello et al., 2017). Although histamine may preferentially act on dural vessels (Kilinc et al., 2017), NGF can directly target trigeminal neurons to enhance their responses to ATP *via* P2X3 receptors (D’Arco et al., 2007). Furthermore, for implementation of multiple migraine-related signaling pathways, the model should include G-protein coupled receptors (GPCR), including those for migraine-specific drugs such as triptans and neurotrophins.

In summary, the idea of the meningeal neuro-immune synapse (Giniatullin et al., 2019; Koroleva et al., 2019; Koyuncu Irmak et al., 2019) is supported by the present model, which improves our understanding of the processes underlying peripheral mechanisms generating migraine pain within the concept of trigeminovascular dysfunction (Messlinger, 2009; Edvinsson et al., 2012; Dodick, 2018). The current model can, therefore, serve as a novel tool for further testing the mechanisms of meningeal trigeminal nociception *in silico*.

## Data Availability Statement

The raw data supporting the conclusions of this article will be made available by the authors, without undue reservation, to any qualified researcher. Requests to access the datasets should be directed to rashid.giniatullin@uef.fi.

## Ethics Statement

The animal study was reviewed and approved by The Committee for the Welfare of Laboratory Animals of the University of Eastern Finland and the Provincial Government of Kuopio.

## Author Contributions

AS developed a model. AS, NM, KK, AV, and OG conducted the experiments. AS, RG, FN, MT, and FG analyzed the results. RG, MT, and AN supervised the whole project. All authors reviewed the manuscript.

## Conflict of Interest

The authors declare that the research was conducted in the absence of any commercial or financial relationships that could be construed as a potential conflict of interest.
