# Supplementary figures and images for: Modeling a Nociceptive Neuro-Immune Synapse Activated by ATP and 5-HT in Meninges: Novel Clues on Transduction of Chemical Signals Into Persistent or Rhythmic Neuronal Firing
Source: Front Cell Neurosci. 2020 May 19;14:135. doi: 10.3389/fncel.2020.00135 (PMC7248338; doi:10.3389/fncel.2020.00135)

## Slide 1
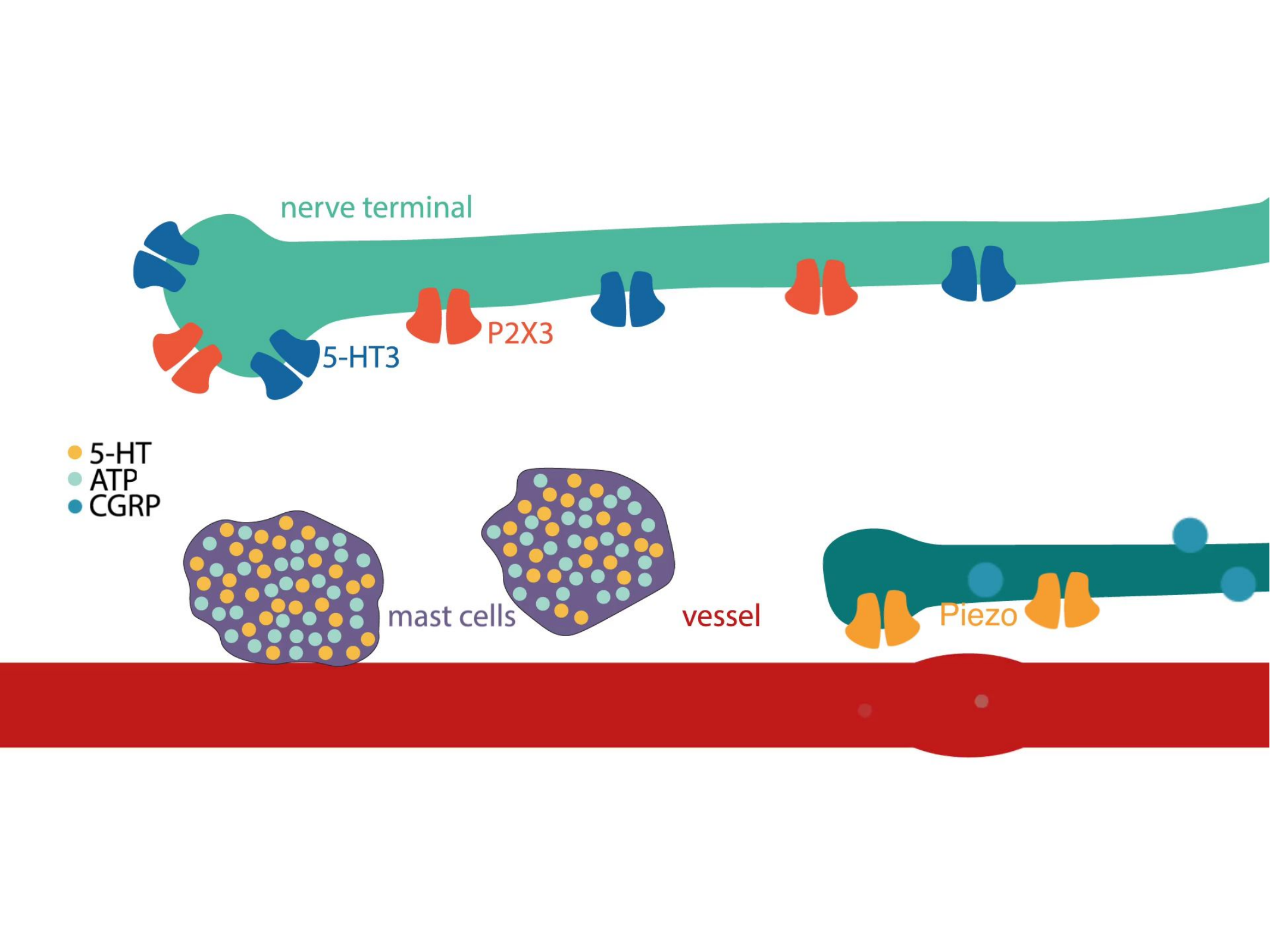

Supplement: Supplementary file 1 [file Presentation_1.PPTX]
