# Supplementary material for: Modeling a Nociceptive Neuro-Immune Synapse Activated by ATP and 5-HT in Meninges: Novel Clues on Transduction of Chemical Signals Into Persistent or Rhythmic Neuronal Firing
Source: Front Cell Neurosci. 2020 May 19;14:135. doi: 10.3389/fncel.2020.00135 (PMC7248338; doi:10.3389/fncel.2020.00135)
Supplement: Supplementary file 2 [file Data_Sheet_1.PDF]

Table 1: The list of ion channels used in the model and their parameters.

Channel type	Conductivity (S/cm^2)	The midpoint of activation (mV)		tau$_{fast}$ (mS)		Reference
Nav1.3	0.2	experiment	—25.5 ± 1.6	experiment	2	(Cummins et al., 2001)
		model	—26	model	2.3	
Nav1.7	0.1	experiment	—20.3 ± 0.8	experiment	0.6	(Tigerholm et al., 2013)
		model	—20	model	0.5	
Nav1.8	0.2	experiment	—1.11 ± 1.6	experiment	5	(Balbi et al., 2017)
		model	—1.1	model	3.9	
K-A	0.1	experiment	—1.4 ± 1.4	experiment	—	(Gasparini et al., 2004)
		model	—1	model	—	
K-DR	0.01	experiment	13.4 ± 1.8	experiment	—	(Tigerholm et al., 2013)
		model	13	model	—	

Table 2: The list of ATP-gated P2X2 and P2X3 and 5-HT-gated 5-HT3 receptors and their parameters

Receptor type	E_v (mV)	Desensitization rate (s)	Recovery time (min)	EC50 (μM)	Reference
P2X2	—6.37±1.09	extremely slow	—	9.8	(Moffatt and Hume, 2007)
P2X3	1 ± 2	24.5 ± 1	2 — 8	1.5	(Sokolova et al., 2006)
5-HT3	28	0.46 ± 0.15	0.25 — 0.5	2.7 ± 0.5	(Corradi et al., 2009)

Table 3: The list of models with their significant parameters where the difference between previous and next model is emphasized.

Model	Degradation coefficient	Conductance of Nav1.7/Nav1.8 channels (S/cm^2)	Number of ATP release events	Topology	Receptors
1	0	0.1 / 0.0	1	line	p2x3
2	**0.001**	0.1 / 0.0	1	line	p2x3
3	**0.1**	0.1 / 0.0	1	line	p2x3
4	0	0.1 / 0.0	1	line	**p2x2**
5	1	0.1 / 0.0	1	line	**p2x2/p2x3**
6	1	**0.2 / 0.0**	1	line	p2x3
7	1	**0.1 / 0.1**	1	line	p2x3
8	1	**0.1 / 0.2**	1	line	p2x3
9	1	0.1 / 0.0	**2**	line	p2x3
10	1	0.1 / 0.2	1	**branching**	p2x3
11	1	0.1 / 0.2	**2**	branching	p2x3

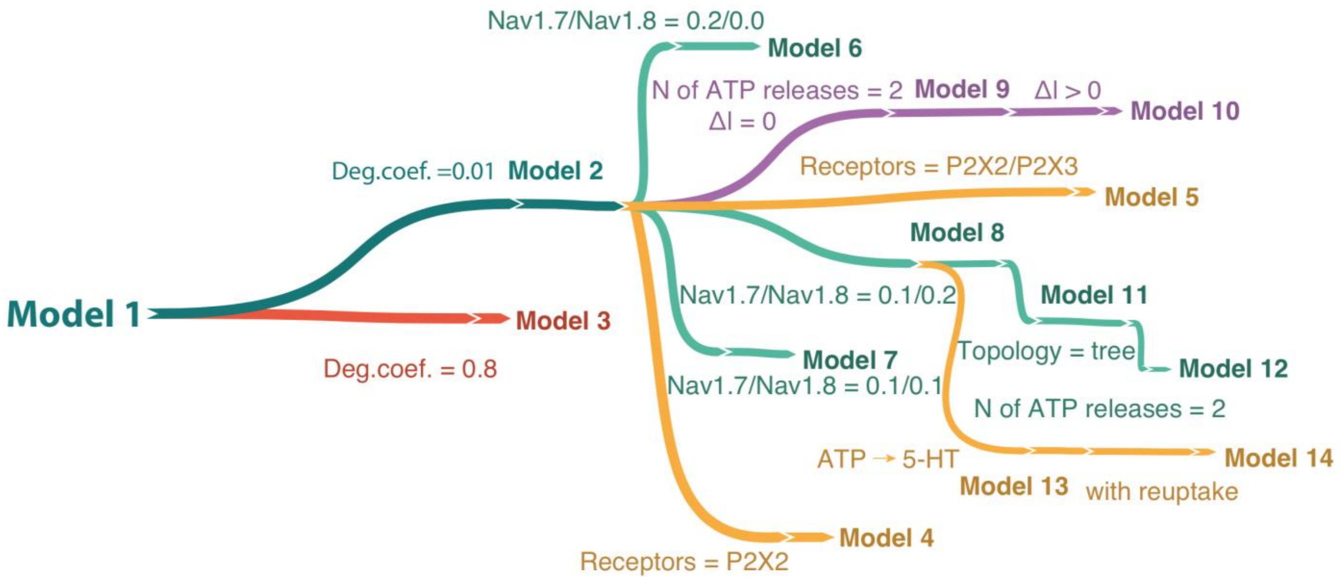

Supplementary Figure 1. The hierarchy of models.

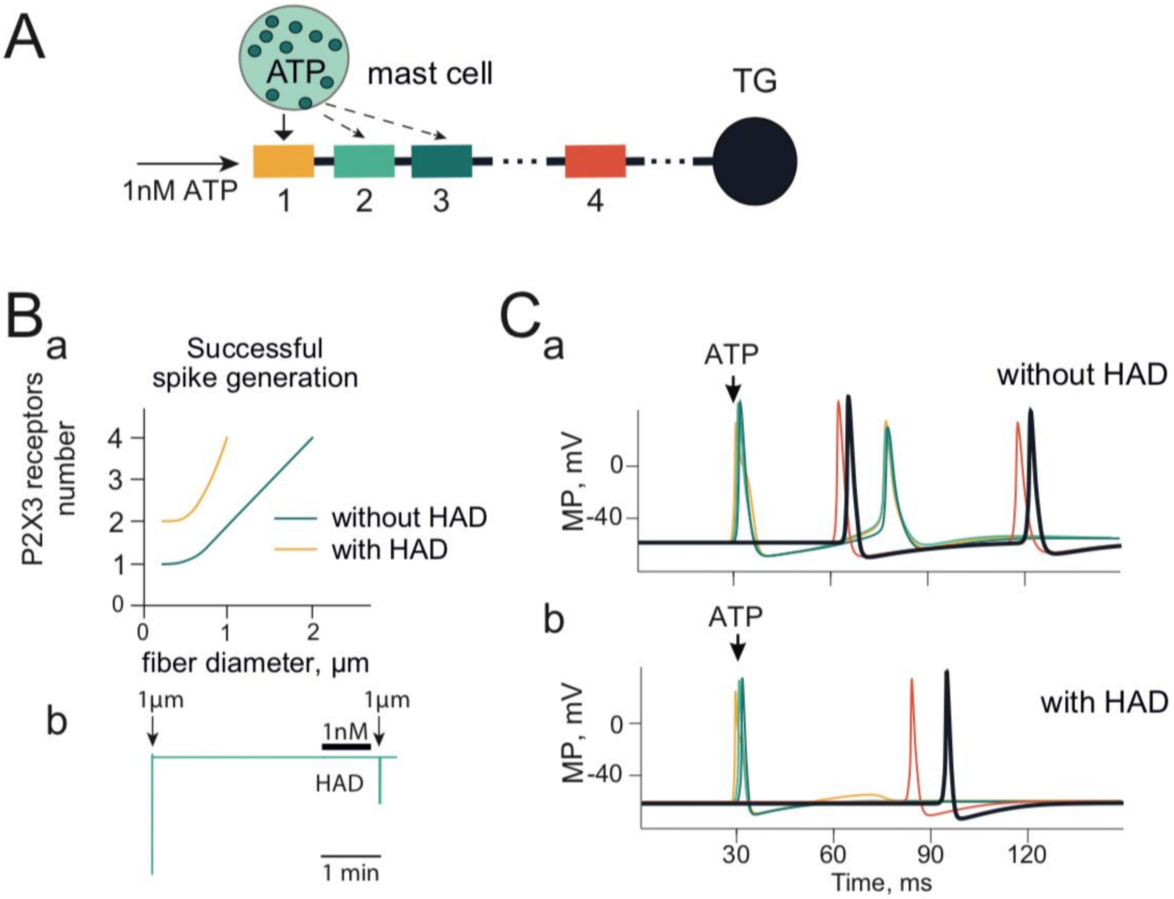

Supplementary Figure 2. Simulation of the role of high-affinity desensitization (HAD) on spiking. (A) Schematic presentation of model with ATP release (1 μM), nerve fiber and HAD effect evoked by 1 nM ATP. (Ba) Graph showing the difference between the number of P2X3 receptors required to trigger a spike with HAD and without HAD. Notice the changed slop of this dependence after HAD. (Bb) Influence of HAD (see 1 nM ATP indicated by horizontal black bar) on the amplitude of P2X3 receptor currents (downward green deflections). (Ca) Spiking activity without HAD or (Cb) with HAD.
